# The role of flavin-containing enzymes in mitochondrial membrane hyperpolarization and ROS production in respiring *Saccharomyces cerevisiae* cells under heat-shock conditions

**DOI:** 10.1038/s41598-017-02736-7

**Published:** 2017-05-31

**Authors:** Irina V. Fedoseeva, Darya V. Pyatrikas, Alexei V. Stepanov, Anna V. Fedyaeva, Nina N. Varakina, Tatyana M. Rusaleva, Gennadii B. Borovskii, Eugene G. Rikhvanov

**Affiliations:** Siberian Institute of Plant Physiology and Biochemistry, 132 Lermontov St., Irkutsk, 664033 Russia

## Abstract

Heat shock is known to accelerate mitochondrial ROS production in *Saccharomyces cerevisiae* cells. But how yeast mitochondria produce ROS under heat-shock condition is not completely clear. Previously, it was shown that ROS production in heat-stressed fermenting yeast cells was accompanied by mitochondrial membrane potential (MMP) increase. In the current investigation the relationship between ROS production and MMP was studied in respiring yeast cells in stationary phase, using diphenyleneiodonium chloride (DPI), an inhibitor of flavin-containing proteins, as well as the mutants deleted for *NDE1*, *NDE2* and *NDI1* genes, encoding flavin-containing external and internal NADH dehydrogenases. It was shown that heat shock induced a transient burst in mitochondrial ROS production, which was paralleled by MMP rise. ROS production and MMP was significantly suppressed by DPI addition and deletion of *NDE1*. The effect of DPI on ROS production and MMP rise was specific for respiring cells. The results obtained suggest that the functioning of mitochondrial flavin-binding enzymes, Nde1p for instance, is required for the hyperpolarization of inner mitochondrial membrane and ROS production in respiring *S. cerevisiae* cells under heat-shock conditions.

## Introduction

Reactive oxygen species (ROS), such as superoxide anion (O_2_^•^−), hydrogen peroxide (H_2_O_2_) and hydroxyl radical (OH^•^) are an unavoidable by-products of oxygen metabolism. In low concentration, ROS serve as signal leading to the activation of gene expression. But in high concentration, ROS are harmful to cells due to their damaging effect on lipids, proteins and nuclear acids. As a rule, ROS level is strictly controlled by activity of antioxidant enzymes, but such balance is disturbed under different pathophysiological situations which leads to the oxidative stress.

Mitochondria can be a major source of ROS generation in eukaryotic cells^1–3^. Mitochondrial ROS production is increased under environmental stimuli and can dramatically affect the pro-survival or pro-death pathways^4, 5^. It is generally accepted that complexes I and III are the main sites of mitochondrial ROS production^1–3^. But this conclusion is based mainly on studies using isolated mitochondria under non-physiological conditions, such as the presence of respiratory inhibitors (rotenone and antimycin A) or when ADP is exhausted (state IV respiration). Natural substances that could mimic the action of respiratory inhibitors remain unknown. Hence, there is a real suspicion that in living cells these complexes make little contribution to ROS production^6^.

In contrast to most eukaryotes, *S. cerevisiae* cells lack the complex I^7^. As an alternative, *S. cerevisiae* cells were shown to contain three rotenone-insensitive NADH:ubiquinone oxidoreductases, which oxidize NADH by reducing ubiquinone without pumping protons across the inner mitochondrial membrane. Internal NADH dehydrogenase (Ndi1p) faces the matrix side and catalyzes the oxidation of NADH generated inside the mitochondria^8^. Two external NADH dehydrogenases (Nde1p and Nde2p) are located on the exterior face of the inner mitochondria membrane and are involved in the oxidation of NADH produced in the cytosol^9–11^.

It was shown^12–15^ that *S. cerevisiae* external and internal dehydrogenases are potential sites of ROS production. Diphenyleneiodonium, an inhibitor of flavin-containing enzymes, inhibited hydrogen peroxide formation in isolated mitochondria supplied by exogenous NADH suggesting that external NADH dehydrogenases produce ROS in *S. cerevisiae* mitochondria under resting conditions^12^. Deletion of external mitochondrial NADH dehydrogenase genes (*NDE1* and *NDE2*) has been reported to reduce the heat-induced ROS production in *S. cerevisiae* cells and mitochondria^13^. Moreover, *NDE1* deletion promoted a decrease in the rate of H_2_O_2_ formation in isolated yeast mitochondria under resting conditions^14^. In agreement with this notion the overexpression of either *NDI1* or *NDE1* caused a significant increase in ROS production^15^. But the role of external and internal NADH dehydrogenases in ROS production is not completely straightforward. There are conflicting results concerning the effect of *NDE1* or *NDI1* deletions on ROS production. It was shown that fermenting *nde1*Δ mutant cells produced elevated levels of superoxide radicals^16^. Mitochondria isolated from *ndi1*Δ mutant released more H_2_O_2_ compared to that of parent type cells^14^. In contrast there was no difference in superoxide radical formation between parent type and *nde1*Δ and *ndi1*Δ mutants in aging *S. cerevisiae* cells^17^. Nde1p, Nde2p and Ndi1p are flavin-containing proteins. Apart from them, there are 36 different flavoproteins operating in the yeast mitochondria. Many of these are directly participating in redox reactions connected to the electron transport chain^18^, suggesting their involvement in ROS production.

Recently we have shown that treatment by moderate heat shock led to progression of ROS-dependent cell death in fermenting *S. cerevisiae* grown on glucose-containing medium. Heat shock induced the ROS production and hyperpolarization of inner mitochondrial membrane. There was a close correlation between these parameters. All agents suppressing the mitochondrial membrane potential (MMP) rise also suppressed ROS production and simultaneously increased yeast thermotolerance, suggesting that generation of ROS and progression of cell death under moderate heat shock are driven by the MMP^19^. Glucose-grown *S. cerevisiae* cells produced more ROS, as compared to respiring cells^3^, but such cells obtain energy mainly by fermentation, so their main mitochondrial functions are repressed. For instance, the functioning of Nde1p and Nde2p is strictly dependent on the availability of cytosolic NADH. The cytosolic NADH/NAD^+^ ratio is neutral in fermenting cells and increases under respiratory growth conditions. Respectively, expression of *NDE1*, *NDE2* and *NDI1* genes are repressed in glucose-grown cells and activated after a diauxic shift^7^. Therefore, it may be expected that a mechanism determining mitochondrial ROS production and MMP maintenance depends on yeast energetic metabolism. Thereby, the question arises whether the link between increased MMP and enhanced ROS production would be valid for respiring yeast cells? And if so, how does heat shock trigger MMP rise in the mitochondria? To answer on these questions we have measured the ROS production, MMP value and thermotolerance in parent type *S. cerevisiae* cells after treatment by diphenyleneiodonium chloride (DPI), an inhibitor of flavin-containing proteins, as well as in mutants deleted for *NDE1*, *NDE2* and *NDI1* genes. Cells were used in stationary phase, when glucose in the growth medium was exhausted and the yeast began to use ethanol due to activation of oxidative phosphorylation.

## Results

### ROS production and hyperpolarization of inner mitochondrial membrane are dependent phenomena in heat-shocked respiring *S. cerevisiae* cells

In our previous work^19^ it was found that moderate heat shock induced a simultaneous burst in ROS production and MMP increase in log-phase *S. cerevisiae* cells growing on glucose. Under these conditions respiration is repressed due to the Crabtree effect. But in the stationary phase, when glucose was exhausted, the yeast cells activate the respiration to use ethanol for ATP synthesis^20^. To verify whether the heat-induced ROS production and MMP rise were occurred in respiring yeast cells entering the stationary phase, cells grown in YEPD medium for 24 h were treated at 45 °C during 0, 10, 30 and 60 min and ROS production and the MMP value were determined by using 2′, 7′-dichlorofluorescein diacetate (H_2_DCF·DA) and MitoTracker Orange (MO), respectively. In each case, the dyes were added to incubation medium during last 10 min.

**Figure 1:**
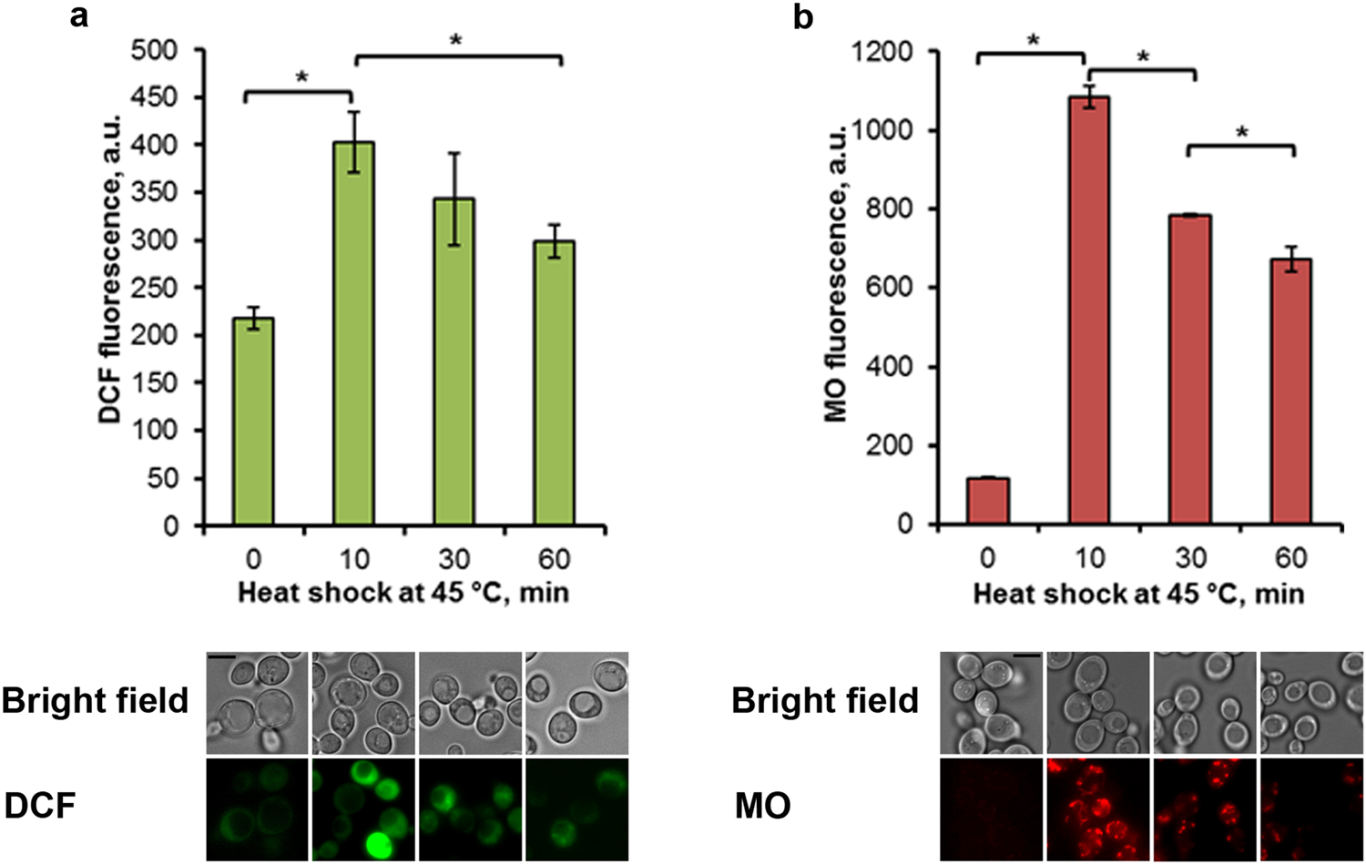
Temporal dynamics of ROS accumulation and MMP rise under heat shock. Cells of strain W303-1A were grown in YEPD medium for 24 h and incubated at 45 °C during 0, 10, 30 and 60 min. DCF (**a**) and MO (**b**) fluorescence were measured immediately after treatment. The data are the means of three independent experiments ± SE. *p < 0.05 (Student’s two-tailed t-test). Microphotographs of yeast cells stained by DCF or MO are presented. Scale bar is 5 μm.

As shown in Fig. 1a, heat treatment induced an increase in DCF fluorescence in the parent strain after 10 min of treatment. Then, DCF fluorescence was declined despite the ongoing heat shock. Change of DCF fluorescence correctly reflected the rate of ROS production in yeast cells under heat-shock condition, because the addition of the antioxidant ascorbic acid effectively suppressed the heat-induced increase in DCF fluorescence^19^. MO fluorescence was found to change in a similar way (Fig. 1b). After 10 min of treatment at 45 °C a clear increase in MO fluorescence was observed. But MO fluorescence was significantly decreased after 30 min of treatment and continued afterwards. The MO fluorescence corresponds to a change in MMP level, since the increase in MO fluorescence was diminished by protonophore addition (see later). Therefore, moderate heat shock at 45 °C induced a transient burst in ROS production in respiring *S. cerevisiae* cells, which was paralleled by a rise in MMP.

Having established that maximal ROS production was observed after 10 min of heat exposure, the dependence of the rate of ROS production and MMP level on the intensity of heat shock was studied. Cells of the parent type were treated at 45 or 50 °C for 10 min and rates of ROS and MMP were measured. As evidenced by the results (see Supplementary Fig. S1), moderate heat shock at 45 °C produced a more profound increase in ROS production (a) and MMP value (b), as compared with severe heat shock at 50 °C. Hence, maximal ROS production and MMP increase were observed, when cells were treated by moderate heat shock at 45 °C, while a further increase of temperature inhibited both processes.

**Figure 2:**
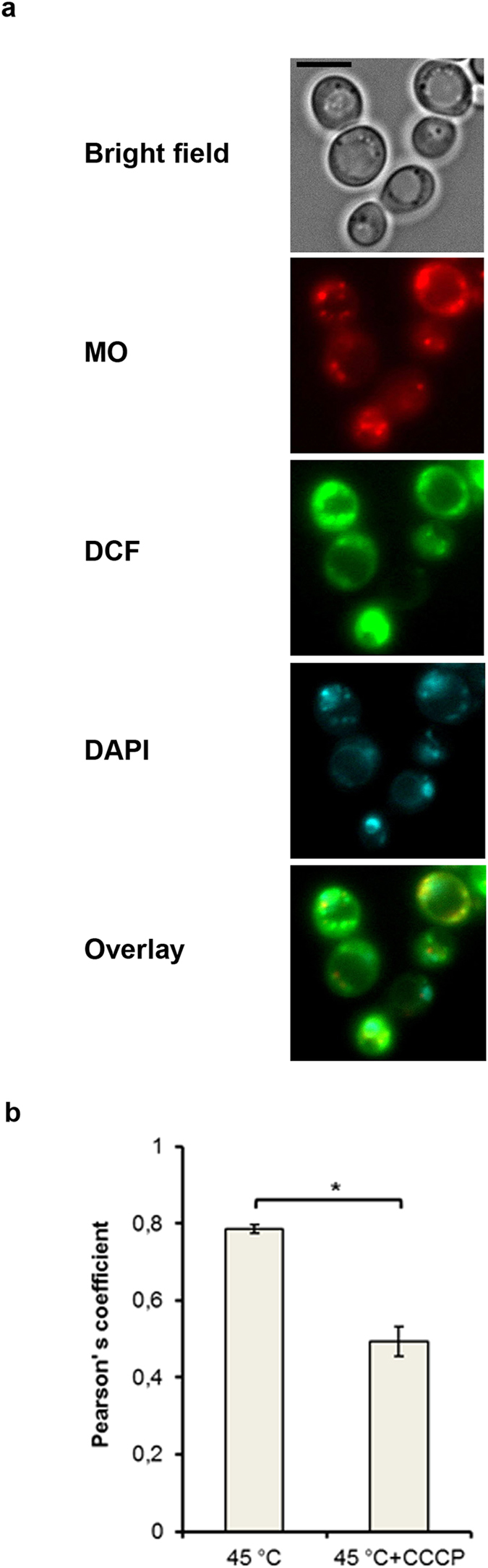
ROS production induced by heat shock is mediated by mitochondria. Cells of strain W303-1A were grown in YEPD medium for 24 h, stained with MO, DCF and DAPI (4,6-diamidino-2-phenylindole) and treated at 45 °C for 10 min. (**a**) Microphotographs of yeast cells are presented. Scale bar is 5 μm. (**b**) The Pearson’s correlation coefficient between MO and DCF fluorescence in control and CCCP-treated cells at 45 °C for 10 min is presented (n = 10 ± SE). *p < 0.05 (Student’s two-tailed t-test**)**.

A parallel change in ROS production and MMP value suggests that mitochondria are the source of ROS. In order to verify it, parent type cells were double-stained with MO and DCF, also 4′, 6-diamidino-2-phenylindole (DAPI) was used to label mitochondrial DNA. The results obtained revealed that DCF fluorescence was partially co-localized with DAPI and MO fluorescence (Fig. 2) indicating that ROS are produced within or around yeast mitochondria. As a rule, cells with higher MO fluorescence had a higher level of DCF fluorescence, confirming a positive correlation between ROS production and inner membrane hyperpolarization. However, in some cells DCF fluorescence was observed without visible MO staining (see Supplementary Fig. S2). Therefore, the population of yeast mitochondria in relation to MMP rise is heterogeneous. In most cases the heat-induced ROS production was initiated in highly polarized mitochondria, but in some cells was not.

**Figure 3:**
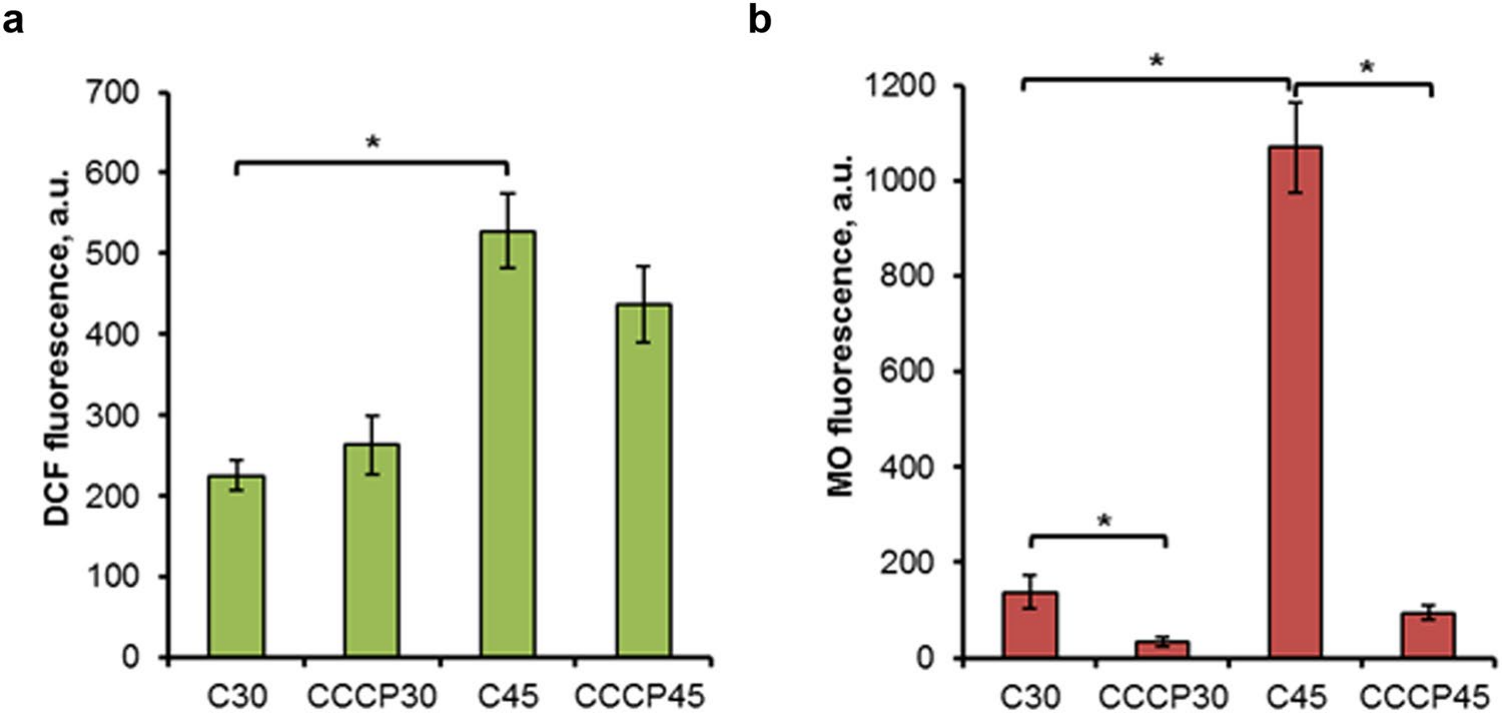
The effect of CCCP on ROS generation and MMP rise under heat shock. Cells of strain W303-1A were grown in YEPD medium for 24 h and incubated at 30 or 45 °C for 10 min in the absence (C, control) or in the presence of 2 μM CCCP. DCF (**a**) and MO (**b**) fluorescence were measured immediately after treatment. The data are the means of four independent experiments ± SE. *p < 0.05 (Student’s two-tailed t-test).

Previously we showed that protonophores, which dissipate the proton gradient on the inner mitochondrial membrane, readily suppress the ROS production in heat-shocked *S. cerevisiae* cells growing on glucose^19^. To investigate whether the same phenomenon was observed in respiring cells, parent type cells were heat-shocked at 45 °C for 10 min in the presence of the protonophoric uncoupler carbonylcyanide *m*-chlorophenylhydrazone (CCCP). Addition of 2 µM CCCP completely inhibited the heat-induced MMP rise (Fig. 3b). But the CCCP effect on ROS production was different. In all experiments performed the addition of CCCP reproducibly suppressed ROS production under heat shock conditions, but the effect was quite small and the final calculation of means of all independent experiments did not show a significant difference (Fig. 3a).

### Heat stress induces the disorganization of the mitochondrial network

Mitochondria are highly dynamic organelles that constantly fuse and divide, forming either interconnected mitochondrial networks or separated fragmented mitochondria. These processes are believed to provide a mitochondrial quality control system and enable an effective adaptation of the mitochondrial compartment to the metabolic needs of the cell^21–23^. It was shown that exposure of fermenting *S. cerevisiae* cells to heat shock led to changes in mitochondrial morphology, as a tubular network disintegrated into several fragmented vesicles^24, 25^. On the other hand, the result obtained by Pozniakovsky *et al*.^26^ suggests that thread-grain transition (mitochondrial fragmentation) occurs in amiodarone treated yeast cells after MMP elevation and subsequent ROS formation.

**Figure 4:**
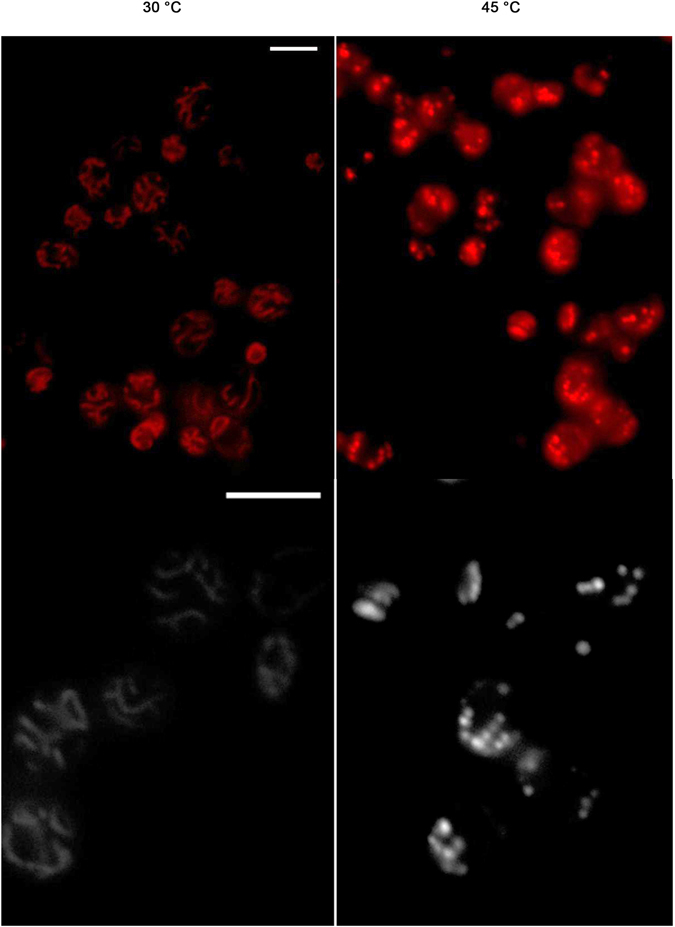
Fragmentation of the mitochondrial network is induced by heat shock. Cells of strain W303-1A were grown in YEPD medium for 24 h, stained with MitoTracker Orange and incubated at 45 °C for 10 min. Microphotographs of yeast cells in pseudo color and gray scale are presented. Scale bar is 5 μm. 100% of yeast mitochondria changed their morphology under heat-shock conditions.

To study whether an increase in MMP and ROS production under heat-shock conditions is accompanied by a change in mitochondrial morphology, parent type cells were stained by MO to label the MMP. As shown in Fig. 4, the control yeast cells maintained a branched tubular mitochondrial network. But in cells treated at 45 °C for 10 min, the disorganization of the mitochondrial network was observed. Mitochondria disintegrated into several fragmented vesicles dispersed throughout the cell volume. Therefore, the heat-induced increase in ROS production and hyperpolarization of inner mitochondrial membrane was accompanied by fragmentation of the mitochondrial network.

### Diphenyleneiodonium chloride inhibits heat-shock induced ROS production and MMP rise, but does not protect the yeast cell from thermal death

Diphenyleneiodonium chloride (DPI), an inhibitor of flavin-containing proteins^27^, has commonly been used to inhibit NADPH oxidases. For instance, yeast NADPH oxidase (Yno1p/Aim14p) was shown to participate in ROS production in yeast cells and its activity to be suppressed by DPI^28^. Inhibition would ultimately result from covalent attachment of phenyl radicals to either the flavin cofactor or adjacent amino acid side chains important in catalysis^27^. However, DPI also potently inhibits mitochondrial ROS production^29^. For instance, DPI reduced hydrogen peroxide formation in isolated yeast mitochondria^12^.

**Figure 5:**
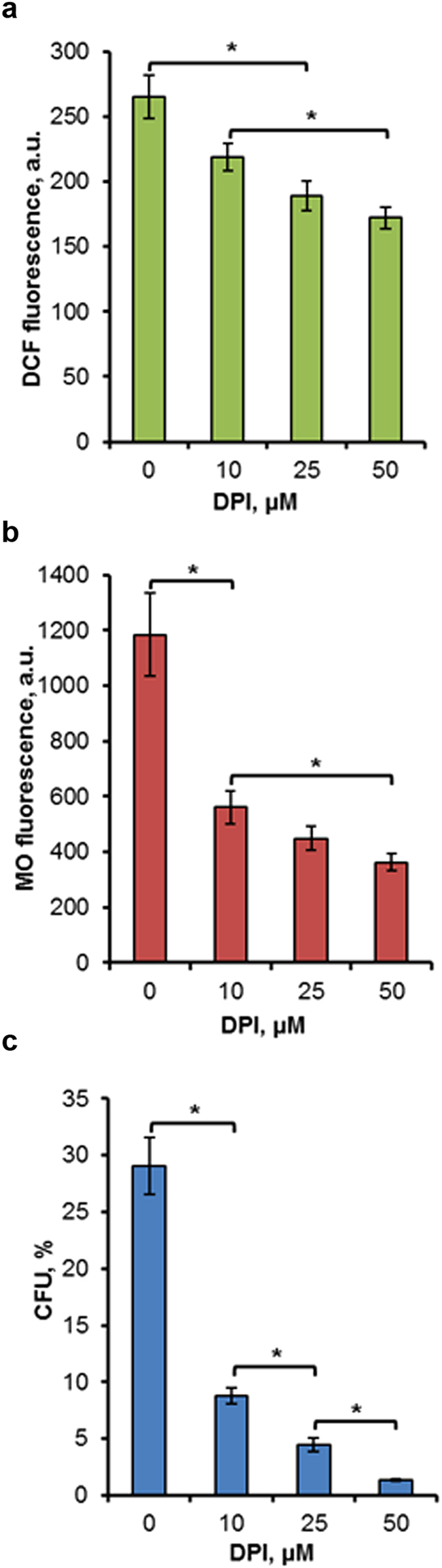
The effect of DPI on ROS generation, MMP rise and cell death under heat shock in parent type cells. Cells of parent type W303-1A were grown in YEPD medium and incubated at 45 °C in the presence of 0, 10, 25 or 50 μM DPI. DCF (**a**) and MO (**b**) fluorescence were measured immediately after 10 min treatment at 45 °C. Survival was evaluated by CFU counting (**c**) after 120 min treatment at 45 °C, followed by 48 h incubation at 30 °C. The data are the means of three or four independent experiments ± SE. *p < 0.05 (Student’s two-tailed t-test).

To study the DPI effect on ROS production and MMP under heat shock, parent type cells grown to stationary phase were treated by 0, 10, 25 and 50 µM DPI and exposed to heat shock at 45 °C for 10 min. As it turned out, 25 µM DPI significantly inhibited heat-shock induced ROS production (Fig. 5a). At the same time, DPI addition suppressed MMP increase after a temperature shift (Fig. 5b), supporting a correlation between mitochondrial hyperpolarization and a burst of ROS production. The fact that DPI simultaneously inhibited ROS production and MMP rise in heat-shocked yeast cells suggests that DPI is a potent inhibitor of mitochondrial ROS production. It is worth noting, that DPI was able to suppress both MMP rise and ROS production in respiring cells only. There was no significant DPI effect on these parameters in fermenting cells (see Supplementary Fig. S3). The latter effect was probably due to the repression of genes encoding mitochondrial flavin-containing enzymes in glucose-grown cells^7^. Hence, the DPI effect on ROS production depends on the energetic metabolism of yeast, supporting the opinion, that DPI primarily suppresses mitochondrial ROS production.

Agents that are able to prevent the heat-induced ROS production, such as glutathione^30^, N-tert-butyl-α-phenylnitrone^31^ and ascorbic acid^19^, as a rule, are capable of increasing yeast thermotolerance. Therefore at the next stage, the effect of DPI on yeast thermotolerance was studied. Parent type cells were treated at 45 °C for 120 min in the presence of DPI. Survival was determined by CFU counting. Treatment at 45 °C led to progression of cell death. DPI addition did not protect parent type cells from thermal death. On the contrary, DPI, in concentration dependent manner strongly decreased the thermotolerance of respiring yeast cells (Fig. 5c). In a similar way, DPI effectively suppressed the thermotolerance of fermenting cells (see Supplementary Fig. S3). Hence, the ability of DPI to suppress the heat-induced ROS production and MMP rise was specific for respiring cells, but its effect on thermotolerance is independent of energetic metabolism. It may be suggested that DPI inhibits some flavin-containing enzymes functioning in both fermenting and respiring cells that are essential for thermotolerance. On the whole, the results obtained suggest that mitochondrial flavin-containing proteins are responsible for the hyperpolarization of inner mitochondrial membrane and ROS production under heat conditions in respiring *S. cerevisiae* cells.

### Deletion of the single *NDE1* gene suppressed the heat-induced ROS generation and MMP increase

**Figure 6:**
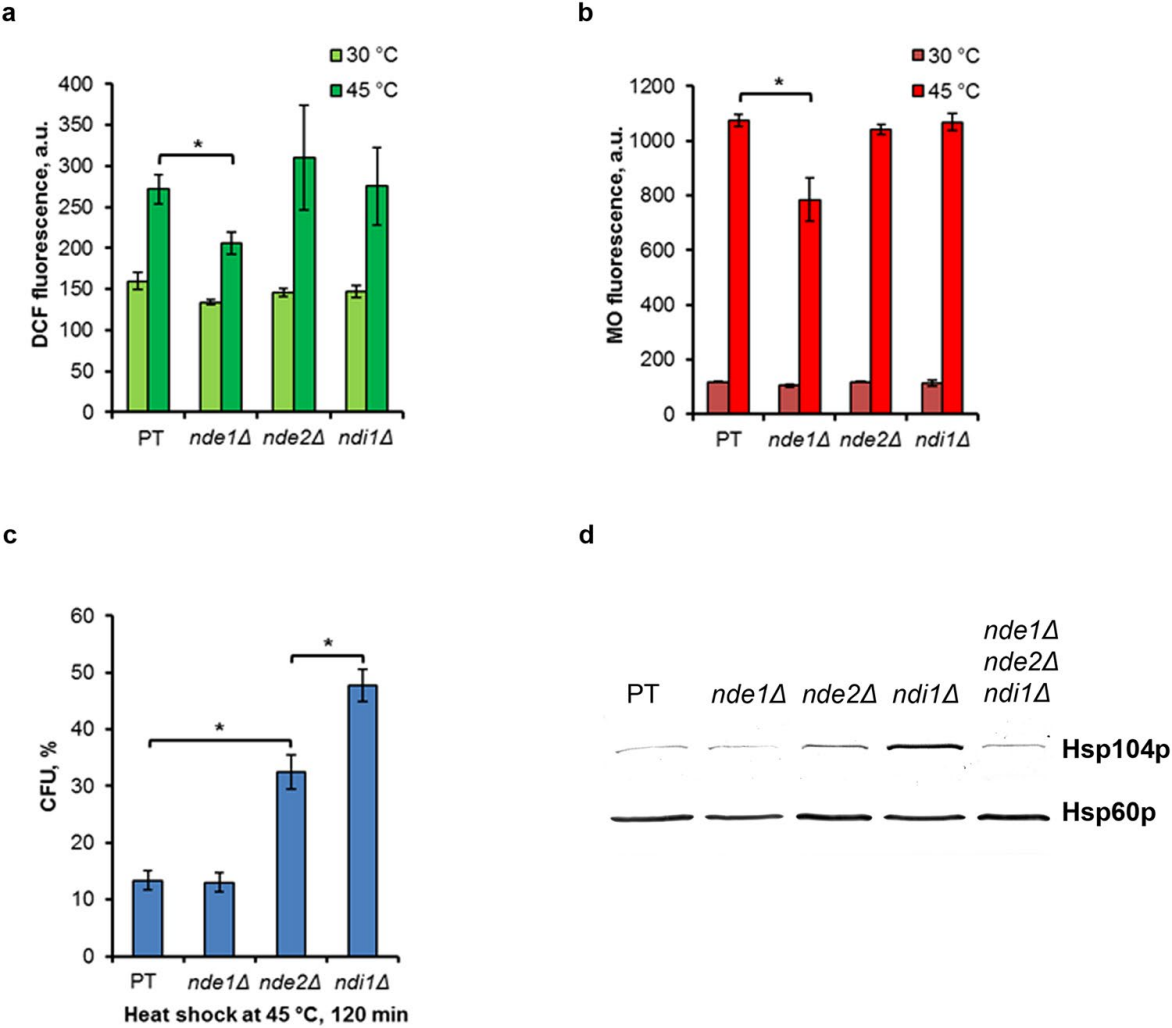
Heat-induced ROS generation, MMP rise, cell death and Hsp104p synthesis in single *nde1*Δ*, nde2*Δ and *ndi1*Δ mutants. Cells of parent type W303-1A (PT), single *nde1*Δ*, nde2*Δ and *ndi1*Δ mutants were grown in YEPD medium and incubated at 45 °C. DCF (**a**) and MO (**b**) fluorescence were measured immediately after 10 min treatment at 45 °C. Survival was evaluated by CFU counting (**c**). The data are the means of three or four independent experiments ± SE. *p < 0.05 (Student’s two-tailed t-test). Immunoblots with anti-Hsp104p and anti-Hsp60p antibodies (**d**). A representative result of three independent experiments was shown.

Previously it was shown that *S. cerevisiae* cells deficient in both external dehydrogenase Nde1p and Nde2p produced a smaller amount of ROS under heat-shock conditions, suggesting that external NADH dehydrogenases are sites for ROS production in stressed yeast^13^. External dehydrogenases Nde1p and Nde2p, as well as, internal dehydrogenase Ndi1p are flavin-containing enzymes^18^. Having shown the possible participation of mitochondrial flavin-containing proteins in ROS production and MMP increase in respiring *S. cerevisiae* cells under heat-shock conditions, we supposed that Nde1p and Nde2p are responsible for both processes. To check this suggestion, mutant cells carrying deletions in external and internal dehydrogenase genes were analyzed. The intensity of DCF and MO fluorescence were compared in parent type and single *nde1*Δ, *nde2*Δ, *ndi1*Δ mutants treated at 45 °C for 10 min. Heat shock increased the ROS production and MMP level in single *nde2*Δ and *ndi1*Δ mutants in a similar way as in parent type (Fig. 6). But cells deleted for *NDE1* produced significantly less ROS (Fig. 6a). Simultaneously, the heat-induced MMP increase was reduced in *nde1*Δ cells (Fig. 6b). These results suggest that, under heat shock conditions, external NADH dehydrogenase Nde1p is involved in ROS production in stationary phase yeast cells, probably via its ability to induce the hyperpolarization of inner mitochondrial membrane.

The thermotolerance of *S. cerevisiae* cells was shown to be dependent on ROS production. Conditions which decrease ROS production, as a rule, increase yeast thermotolerance^19, 32^. Since mutants used in this study displayed a significant difference in ROS production under heat-shock conditions, we determined their thermotolerance to heat shock. Cells of parent type, single *nde1*Δ, *nde2*Δ and *ndi1*Δ mutants were grown up to the stationary phase, exposed at 45 °C for 0–240 min and survival was determined. Surprisingly, deletion of *NDE1* (*nde1*Δ mutant), which significantly decreased the heat-induced ROS production (Fig. 6a) did not have any obvious effect on yeast thermotolerance (Fig. 6c). Quite the contrary, *NDI1* deletion (*ndi1*Δ mutant) significantly protected the yeast cells from thermal death. Deletion of *NDE2* (*nde2*Δ mutant) also led to protection from moderate heat shock at 45 °C, but its effect was rather less (Fig. 6c). Thus, we did not observe a correlation between ROS production and yeast thermotolerance in the stationary phase culture. It was previously shown that *NDI1* deletion protects yeast cells from death induced by hydrogen peroxide, but its effect is separable from the NADH dehydrogenase activity^33^.

The heat shock protein 104 (Hsp104p) plays a key role in thermotolerance in *S. cerevisiae* cells. Level of Hsp104p was found to be elevated in stationary phase which accompanied by increase of thermotolerance^34^. As expected, the constitutive level of Hsp104p synthesis was rather high in stationary phase parent type cells without additional heat treatment (Fig. 6d). Level of Hsp104p synthesis in mutant cells lacking Nde1p (*nde1*Δ mutant) was the same as in parent type, but Hsp104p level was elevated in cells deficient in Nde2p and Ndi1p. It was observed a close correlation between thermotolerance and Hsp104p synthesis. *ndi1*Δ mutants cells were highly resistant to the damaging effect of heat shock (Fig. 6c) and synthesized more Hsp104p (Fig. 6d).

To verify the role of external and internal NADH dehydrogenases in ROS production under heat-shock conditions a similar experiment was conducted in log-phase yeast cells growing on galactose which supports a higher rate of respiratory metabolism than does glucose. As shown in Supplementary Fig. S4, the absence of Nde1p decreased the ROS production induced by treatment at 45 °C. Deletion of *NDE2* did not cause a significant effect on ROS production. It is interesting that the loss of *NDI1* also promoted a decrease in the rate of ROS production in galactose grown cell. MMP level in heat-shocked mutant cells was found to change in a similar way (see Supplementary Fig. S4).

**Figure 7:**
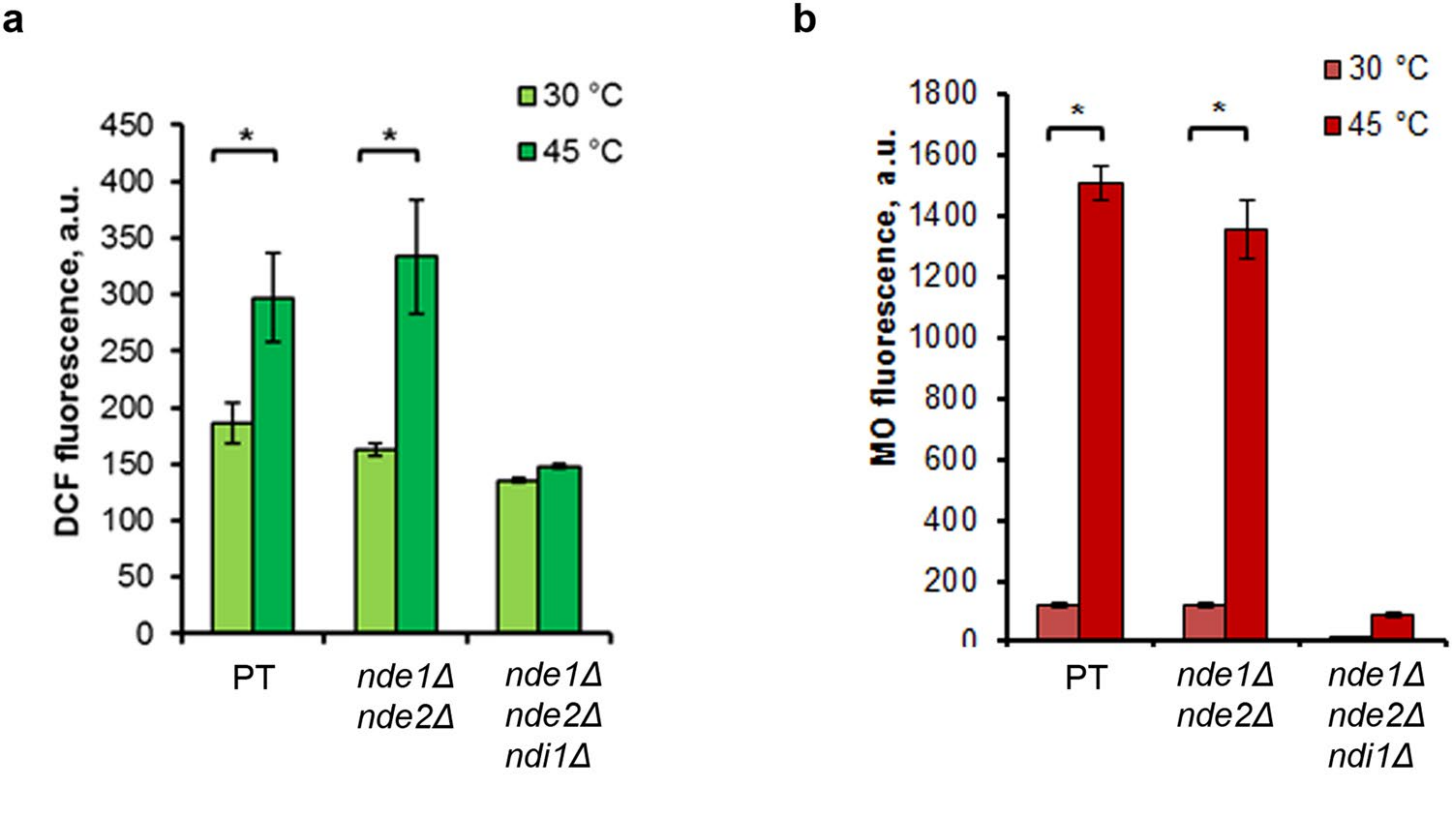
Heat-induced ROS generation and MMP rise in double (*nde1*Δ*nde2*Δ) and triple (*nde1*Δ*nde2*Δ*ndi1*Δ) mutants. Cells of parent type W303-1A (PT), double *nde1*Δ*nde2*Δ and triple *nde1*Δ*nde2*Δ*ndi1*Δ mutants were grown in YEPD medium and incubated at 45 °C for 10 min. DCF (**a**) and MO (**b**) fluorescence were measured immediately after treatment. The data are the means of three or four independent experiments ± SE. *p < 0.05 (Student’s two-tailed t-test).

In a subsequent experiment, ROS production and MMP change were measured in double *nde1*Δ*nde2*Δ and triple *nde1*Δ*nde2*Δ*ndi1*Δ mutants subjected to heat shock. Surprisingly, the stationary phase cells deficient in both external dehydrogenase Nde1p and Nde2p produced the same rate of ROS and MMP under heat-shock conditions, as parent type cells. In contrast, ROS production and MMP rise was completely suppressed in cells of the triple *nde1*Δ*nde2*Δ*ndi1*Δ mutant (Fig. 7a,b). Hence, our results do not support the finding of Davidson & Schiestl^13^, in our case, cells deficient in both external dehydrogenase Nde1p and Nde2p produced the same ROS rate, as the parent type. The contradiction may be due to the fact that a clear effect of double *NDE1* and *NDE2* deletion on ROS production was obtained by Davidson & Schiestl^13^ in the case of isolated mitochondria or when using a parent strain with different genetic background (JM43). Nevertheless our data supports the conclusion^12–14^ that external NADH dehydrogenases participate in ROS production in yeast cells. Moreover, the decrease in ROS production in the *nde1*Δ mutant was accompanied by suppression of heat-induced hyperpolarization of the inner mitochondrial membrane. The additional deletion of *NDE2* restores the rate of ROS production and MMP value, probably via activation of other metabolic routes. Deletion of all external and internal NADH dehydrogenases in the triple *nde1*Δ*nde2*Δ*ndi1*Δ mutant completely suppressed the ROS production and MMP rise under heat-shock conditions, probably via generation of *petite* cells without mitochondrial DNA^14^. There was no mitochondrial DNA in the cells of the triple *nde1*Δ*nde2*Δ*ndi1*Δ mutant as revealed by DAPI staining (see Supplementary Fig. S5). Moreover, triple mutant cells failed to use the ethanol, non-fermenting substrate, for growth (see Supplementary Fig. S6). Comparison of ROS production and thermotolerance between triple *nde1*Δ*nde2*Δ*ndi1*Δ mutant and isogenic *petite* mutant did not reveal any difference (see Supplementary Fig. S7).

ROS production and respiration can change in the same or in the opposite directions depending on the redox status of the ROS producing site^1^. On the other hand, the results obtained above suggest a close link between MMP level and ROS production in respiring yeast cells. With this in mind, we determined the respiratory activity of mutant strains by measuring the oxygen consumption at 30 °C. As shown in Supplementary Fig. S8, the single *nde2*Δ, *ndi1*Δ and double *nde1*Δ*nde2*Δ mutants have a similar rate of oxygen consumption to that of the parent type. The single *nde1*Δ mutant displayed a reduced oxygen consumption: about half that of the parent type. There was practically no oxygen consumption in the case of the triple *nde1*Δ*nde2*Δ*ndi1*Δ mutant. Hence, a decrease in respiratory activity in the yeast strains studied is accompanied by a decrease in heat-induced ROS production and MMP.

### DPI suppresses ROS production and MMP increase under heat-shock conditions in cells deficient in both Nde1p and Nde2p

**Figure 8:**
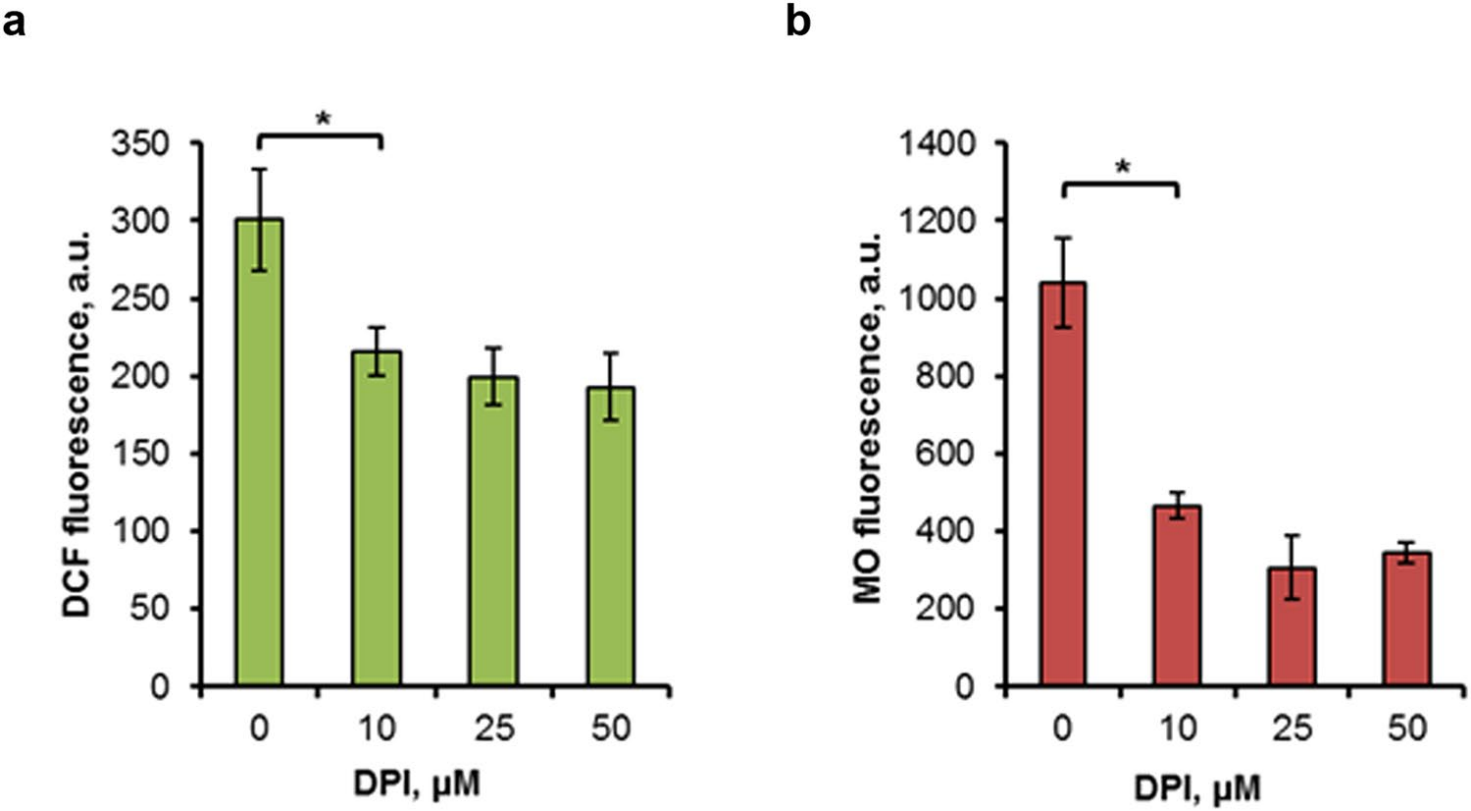
The effect of DPI on ROS generation and MMP rise in double *nde1*Δ *nde2*Δ mutant under heat shock. Cells of *nde1*Δ*nde2*Δ mutants were grown in YEPD medium and incubated at 45 °C for 10 min in the presence of 0, 10, 25 or 50 μM DPI (D). DCF (**a**) and MO (**b**) fluorescence were measured immediately after 10 min treatment at 45 °C. The data are the means of four independent experiments ± SE. *p < 0.05 (Student’s two-tailed t-test).

It was shown that the addition of DPI inhibited hydrogen peroxide formation in yeast mitochondria supplied with NADH as substrate^12^. Authors suggested that external NADH dehydrogenases may function as a site of mitochondrial superoxide production, since complex I is absent in *S. cerevisiae* cells and exogenous NADH cannot cross the mitochondrial membrane. To verify whether the ability of DPI to suppress the heat-induced ROS production and MMP level is due to its ability to inhibit external NADH dehydrogenases, in a subsequent experiment we studied the DPI effect using an *nde1*Δ*nde2*Δ double mutant deleted in both external NADH dehydrogenases. As can be seen in Fig. 8, DPI addition suppressed ROS production (Fig. 8a) and MMP rise (Fig. 8b) in the *nde1*Δ*nde2*Δ mutant under heat-shock conditions in a similar way to that in parent type cells (Fig. 5a,b). Hence, the results of the experiment suggest that the ability of DPI to suppress heat-induced ROS production and MMP in *S. cerevisiae* cells did not depend on external Nde1p and Nde2p dehydrogenases. However it could not be excluded, that other flavin-containing enzymes capable of catalyzing the oxidation of cytosolic NADH are activated in the absence of Nde1p and Nde2p (see Discussion).

## Discussion

Heat shock is known to increase the mitochondrial ROS production in *S. cerevisiae* cells^13, 19, 31, 32, 35^ and such a result was supported by the current study (Figs 1 and 2). As ROS production was shown to be dependent on MMP increase in fermenting *S. cerevisiae* cells^19^, we decided to investigate whether the same phenomenon occurs during respiratory metabolism in stationary cultures and find the mitochondrial electron carriers responsible for mitochondrial hyperpolarization. The results obtained in the course of the current investigation shows a close correlation between ROS production and the hyperpolarization of inner mitochondrial membrane in respiring *S. cerevisiae* cells under heat-shock conditions. Moderate heat shock induced a transient increase in MMP, which was paralleled by a burst in ROS production (Fig. 1). As a rule, ROS production was observed in highly polarized mitochondria (Fig. 2). Elevation of temperature up to 50 °C (see Supplementary Fig. S1) or treatment by DPI (Fig. 5) at 45 °C suppressed MMP increase and simultaneously inhibited the ROS production. Deletion of the external dehydrogenase gene *NDE1* reduced the increase of MMP in heat-shocked *S. cerevisiae* cells (Fig. 6b), which was accompanied by a decrease in ROS production (Fig. 6a). The results obtained strongly suggest that a transient hyperpolarization of the mitochondrial inner membrane drives a ROS production under heat-shock conditions in respiring *S. cerevisiae* cells. However, the protonophore CCCP, which allows a free transport of protons across the inner mitochondrial membrane, thereby dissipating mitochondrial membrane potential, readily suppressed the heat-induced increase in MMP (Fig. 3b), but its effect on ROS production in respiring yeast cells was insignificant (Fig. 3a). On the other hand, CCCP strongly suppressed ROS production under heat shock in fermenting yeast cells^19^.

The proposed link between MMP and ROS production in *S. cerevisiae* cells under heat-shock conditions is in line with the hypothesis proposed by V. P. Skulachev^36^, which postulated that high mitochondrial membrane potential (i.e. mitochondrial hyperpolarization) promotes an increase in ROS production, which in turn is accompanied by so called mild uncoupling (i.e. an increase of H^+^ conductance across the mitochondrial membrane) to prevent the formation of harmful ROS. Accordingly to hypothesis the mild uncoupling might be catalyzed by uncoupling proteins (UCPs). UCPs are members of the superfamily of mitochondrial carriers which transport protons across the inner membrane thereby dissipating the MMP. Indeed, the results obtained by studying the UCPs functions revealed that these proteins decreased MMP and simultaneously reduced mitochondrial ROS production^37^. For instance, the overexpression of mammalian *UCP1* in yeast cells carrying the *RAS2* ^*val19*^ mutation decreased the mitochondrial ROS production^38^.

Since mitochondrial ROS production was shown to increase under a variety of pathophysiological conditions^3^, Skulachev’s hypothesis predicts that in all these cases the hyperpolarization of mitochondrial membrane should occur and be the cause of increased ROS production. Actually, there are some examples showing that treatment of yeast cells with isoprenoid farnesol^39^, acetic acid^40, 41^, hydrogen peroxide^42^, α-factor^43^, amiodarone^26^, cadmium^44^, or grapefruit seed extract, which has anti-fungal activity^45^ induced a hyperpolarization of inner mitochondrial membrane, which was accompanied by increased ROS production. The same situation was observed in *S. cerevisiae* cells in the case of heterologous expression of human Bax^46^, mutation *RAS2* ^*val19*^ ^38^, deletion of *HXK2* ^41^, deletion of *GUP1* ^47^ and mutations which reduce TOR signaling^48^. It seems that the same rule is valid for heat-shock conditions as well. The heat-induced hyperpolarization of inner mitochondrial membrane was shown to occur in mammalian^49–51^, plant^52–54^ and yeast cells^19^. There are some examples showing that heat-induced ROS production is accompanied by MMP increase^50, 51, 54^. Previously, we had shown that there is a strong correlation between MMP increase and ROS production in fermenting *S. cerevisiae* cells^19^. Now we have shown that the same phenomenon is observed in respiring *S. cerevisiae* cells, i.e. heat shock induces an increase in MMP, which supposedly drives mitochondrial ROS production.

Despite some reports supporting Skulachev’s hypothesis (i.e. hyperpolarization drives an increased ROS production), some authors believed that MMP decrease (but not increase) is the cause of ROS production in *S. cerevisiae* cells. Deletions of *ATG1*, *ATG6*, *ATG8* and *ATG12 S. cerevisiae* genes (autophagy-related genes) reduced MMP, but increased ROS production^55^. A decrease in actin dynamics causes a loss of MMP and an increase in ROS production^56, 57^. Treatment of yeast cells by formic acid induces ROS production and loss of MMP^58^. Chronic exposure of yeast cells to the uncoupler FCCP accelerates mitochondrial ROS formation^59^. A prolonged heat treatment at 42 °C (48 h) led to a decrease in the MMP and increase in ROS formation in yeast cells^60^.

To explain the contradictory relationship between MMP value and ROS production, it must be stressed that heat-induced hyperpolarization of inner mitochondrial membrane is a very transient phenomenon in respiring yeast cells. It was observed only after 10 min of heat exposure, after that MMP was shown to decline (Fig. 1). It appears a similar sequence of events was triggered under other pathophysiological conditions. Treatment by acetic acid^40^, amiodarone^26^, grapefruit seed extract^45^ induced an initial rise in MMP and ROS production, which was followed by a decline in both parameters. Therefore there is a possibility that some researchers observing a concomitant MMP decrease and ROS production simply missed the point when hyperpolarization was replaced by depolarization.

According to Skulachev’s hypothesis, if mild uncoupling, for some reason, is unable to prevent ROS formation, then the mitochondrial permeability transition pore (PTP) will be opened. PTP is an increase in mitochondrial inner membrane permeability to solutes with molecular masses up to about 1500 Da. The PTP opening results in swelling of the mitochondrial matrix, disruption of the outer mitochondrial membrane, MMP collapse and release of mitochondrial proteins^36^. Moreover PTP could dramatically increase ROS production^61^. PTP can be observed also in yeast mitochondria^62^. It was supposed that mitochondrial thread-grain transition (mitochondrial fragmentation) precedes PTP opening^63^. Mitochondrial fragmentation was observed in yeast cells treated by amiodarone, which was preceded by an increase in MMP and ROS production, followed by MMP decline^26^. A similar sequence of events was observed in the current investigation. The initial increase in MMP and ROS production in heat-shocked cells (Fig. 1) was accompanied by fragmentation of the mitochondrial network (Fig. 4) and MMP decline (Fig. 1) suggesting that PPT opening was initiated after the initial rise of MMP. The ambiguous effect of CCCP on ROS production (Fig. 3a) and the appearance of cells in which ROS production occurred in depolarized mitochondria (see Supplementary Fig. S2) supports this point of view. Hence, a link between MMP and ROS production may be direct during the initial stage of stress exposure or reversed during the advanced phase.

Another explanation of conflicting results concerning the relationship between MMP and ROS is that depolarization of mitochondrial membrane under some stress conditions might be due to disruption of terminal mitochondrial electron carriers leading to overreduction of upstream segments of the respiratory chain, making them prone to ROS formation. It is well known that rotenone and antimycin A, inhibitors of complex I and complex III, respectively, induce a massive ROS production in cells and isolated mitochondria, despite their ability to reduce MMP^1–3^. In addition, defects in the electron transport chain could lead to ROS production in other cellular compartments. For instance, the inactivation of the yeast cytochrome oxidase complex correlates with ROS generation in ER mediated by ER-localized NADPH oxidase Yno1p^64^.

The main goal of the current investigation was to decipher the mechanism of heat-shock induced mitochondrial hyperpolarization in *S. cerevisiae* cells, leading to an increase in ROS production. The ability of DPI to suppress the increase of MMP and ROS production during heat shock (Fig. 5) suggests that mitochondrial flavin-containing proteins are responsible for both processes. External (Nde1p and Nde2p) and internal (Ndi1p) mitochondrial NADH dehydrogenases are flavin-containing proteins^18^. It was shown previously that Nde1p, Nde2p and Ndi1p are involved in ROS production^12–15^. The results obtained partly support this point of view, deletion of *NDE1* led to suppression of heat-induced ROS production (Fig. 6a) indicating that Nde1p is responsible for mitochondrial ROS production under heat-shock conditions. Moreover, because the increase in MMP induced by heat shock was reduced in the *nde1*Δ mutant (Fig. 6b) it becomes evident, that mitochondrial hyperpolarization was mediated by Nde1p. But Nde1p is not the only player capable of mediating an increase in MMP and ROS production under heat-shock conditions. The loss of Nde1p resulted in only partial inhibition of MMP rise and ROS production (Fig. 6a,b). In contrast, DPI almost completely inhibited both processes (Fig. 5a,b), suggesting that other flavin-containing proteins are involved in MMP buildup and ROS production. It could not be excluded that DPI suppressed the ROS production mediated by NADPH oxidase Yno1p. Although Yno1p is localized in ER^28^, its ability to produce ROS depends on mitochondrial activity^64^.

Genome analysis of *S. cerevisiae* identified 68 genes encoding flavin-containing proteins. More than half the yeast flavoproteins operate in the mitochondrion. More than a quarter of the yeast flavoproteins participate in redox reactions in the mitochondrion^18^. There are results showing that flavin-containing proteins, such as the subunit of yeast succinate dehydrogenase, Sdh1p^65^, dihydrolipoamide dehydrogenase, Lpd1p^66^, apoptosis-inducing factor, Aif1p^67^ participated in ROS generation in *S. cerevisiae* cells. Moreover, yeast cells deficient in electron transferring flavoproteins Aim45p and Cir2p displayed reduced ROS production under heat-shock conditions^35^. All these proteins could be involved in ROS production and MMP increase in yeast cells upon temperature elevation and DPI suppresses both processes by inhibiting theirs activity (Fig. 5). DPI is very hydrophobic and carries a delocalized positive charge, allowing the molecule to diffuse across membranes. Because actively respiring mitochondria have a transmembrane potential with the inside negative, the concentration of DPI may be much higher in the matrix than outside mitochondria^68^. Therefore in living cells DPI can inhibit both external mitochondrial NADH dehydrogenases and other flavin-containing proteins localized in mitochondrial matrix.

The question arises why the single deletion of the *NDE1* gene led to suppression of heat-induced mitochondrial hyperpolarization and ROS production (Fig. 6a,b), while such an effect was absent in the case of double *NDE1*/*NDE2* deletion (Fig. 7a,b)? Furthermore, why did DPI suppress MMP and ROS production in the double *nde1*Δ*nde2*Δ mutant in a similar way as in the parent type (Fig. 8)? In this connection it is important to mention that NADH dehydrogenases Nde1p/Nde2p *S. cerevisiae* form a large supramolecular complex containing the glycerol-3-phosphate dehydrogenase Gut2p^69^. Gut2p is also a flavin-containing protein; it participates in the glycerol-3-phosphate shuttle, which is involved in oxidizing excess of cytoplasmic NADH by mitochondria with the concurrent reduction of FAD to FADH_2_^70^. It is known that Gut2p activity is inhibited by Nde1p and Nde2p^71^. Although we have no information about Gut2p involvement in ROS production in *S. cerevisiae* cells, glycerol-3-phosphate dehydrogenase in mammalian tissues was shown to participate in ROS production and such a process is highly MMP dependent^72^. Therefore we speculate that glycerol-3-phosphate dehydrogenase Gut2p becomes involved in MMP increase and ROS production under heat-shock condition in *S. cerevisiae* cells, if the functional NADH dehydrogenases Nde1p and Nde2p are absent.

It is generally accepted that thermotolerance of *S. cerevisiae* cells is dependent on ROS production^19, 32^. But as a rule, such experiments have been performed using fermenting cells. As it turned out the progression of heat-induced cell death in respiring cells under given conditions was independent on ROS production. The loss of Nde1p resulted in a decrease in ROS production (Fig. 6a), but did not affect thermotolerance (Fig. 6c,d). On the contrary, heat-induced ROS production in cells deficient in Nde2p and Ndi1p was the same as in parent type cells (Fig. 6a), but their thermotolerance was significantly increased (Fig. 6c,d). Transition to the stationary phase is known to activate the expression of genes required for oxidative phosphorylation as well as for stress tolerance and antioxidant defense^73^. Therefore we suggest that increased synthesis of antioxidant enzymes, leading to the fast detoxification of harmful ROS, compensate the difference in the ROS-producing capability of respiring yeast cells.

## Materials and Methods

### Yeast strains and growth conditions

*S. cerevisiae* W303–1A strain (*Mat* **a** *ade2-1 ura3-1 his3-11,15 leu2-3,112 trp1-1 can1-100, SUC2*) was used as a parental type strain. Deletion mutants of external and internal mitochondrial NADH dehydrogenases were kindly provided by Professor Mario H. Barros (Departamento de Microbiologia, University of São Paulo, Brazil)^14^. All yeast cells were cultured in YEPD medium (20 g/L peptone, 10 g/L yeast extract and 20 g/L glucose). The cells were grown at 30 °C in 100 ml Erlenmeyer flasks with 25 ml of liquid YEPD with shaking at 150 rpm during 24 h. After that time, cultures reached the stationary phase (see Supplementary Fig. S9).

### Fluorescence microscopy

ROS generation was studied with the use of 50 μM 2′, 7′-dichlorofluorescein diacetate (H_2_DCF·DA). The MMP was qualitatively visualized using the potential-dependent cationic dye MitoTracker Orange (MO) at a final concentration of 50 nM. The mitochondrial network was visualized following 10 min incubation of the cells with MO and 5 μg/ml DAPI (4′, 6-diamidino-2-phenylindole). DCF and MO fluorescence were analyzed with the use of an inverted fluorescent microscope AxioVision Z1 (Germany), a digital monochromic camera AxioCamMRm3 and the software package AxioVision Rel.4.6 designated for image capture and analysis. The intensity of MO and DCF fluorescence was analyzed using ImageJ software package. In total, ten images of each variant were analyzed from at least three independent experiments. The value of intensity was expressed as arbitrary units. The following filters were used: Filter set 10 (EX BP 450–490, BS FT 510, EM BP 565 – for detecting DCF fluorescence), Filter set 20 (EX BP 546/12, BS FT 560, EM BP 575–640 – for detecting MO fluorescence) and Filter set 49 (EX G 365, BS FT 395, EM BP 445/50 – for detecting DAPI fluorescence). Co-localization between DCF and MO fluorescence was assessed using Pearson’s coefficient with help of ImageJ with JACoP plugin^74^.

### Assay of viability

Cells were grown to a stationary growth phase at 30 °C. The 1 ml cell suspensions were transferred to glass tubes and immersed in a shaking water bath at 45 or 50 °C. Portions of the cell suspensions were withdrawn for indicated periods of time and chilled on ice. The effect of diphenyleneiodonium chloride (DPI) on thermotolerance was studied by the addition of this agent to the yeast cells at 30 or 45 °C. To count colony-forming units (CFU), the yeast cells were diluted and plated in a standard way in YEPD medium containing 15 g/L agar. After 24–48 h incubation at 30 °C, the CFU were counted, and the data are represented as a percentage of the control.

### Isolation of total proteins and immunoblotting

The cells were centrifuged, washed thrice with distilled water, and stored at –70 °C before proteins isolation. The cells were thawed, resuspended in isolation buffer (0.1 M Tris HCl, pH 7.4–7.6, containing 3 mM SDS and 1 mM β-mercaptoethanol), frozen in liquid nitrogen, and ground with quartz sand. Crude cell components were removed by centrifugation (15,000 g for 15 min), and the protein was treated with three volumes of cold acetone. The pellet was washed thrice with acetone and dissolved in sample buffer (0.625 M Tris HCl, pH 6.8, containing 8 mM SDS, 0.1 M β-mercaptoethanol, 10% glycerol, and 0.001% bromophenol blue). Protein concentration was determined by the Lowry method. Following SDS-PAGE in 12% polyacrylamide gel, immunoblotting was carried out using antibodies against Hsp104p (SPA 8040; Stressgen, USA) and against Hsp60p (USBiological H1830 77B, USA).

### Reproducibility of the results

All experiments were repeated a minimum of three times. The data obtained were analyzed statistically, i.e., arithmetic means and standard errors were determined. For comparison between two groups the impaired Student’s test was used.

## Electronic supplementary material

The role of flavin-containing enzymes in mitochondrial membrane hyperpolarization and ROS production in respiring Saccharomyces cerevisiae cells under heat-shock conditions
