# Supplementary material for: The role of flavin-containing enzymes in mitochondrial membrane hyperpolarization and ROS production in respiring Saccharomyces cerevisiae cells under heat-shock conditions
Source: Sci Rep. 2017 May 31;7:2586. doi: 10.1038/s41598-017-02736-7 (PMC5451409; doi:10.1038/s41598-017-02736-7)
Supplement: Supplementary file 1 — The role of flavin-containing enzymes in mitochondrial membrane hyperpolarization and ROS production in respiring Saccharomyces cerevisiae cells under heat-shock conditions [file 41598_2017_2736_MOESM1_ESM.pdf]

Supplementary

The role of flavin-containing enzymes in mitochondrial membrane hyperpolarization and ROS production in respiring *Saccharomyces cerevisiae* cells under heat-shock conditions

Irina V. Fedoseeva, Darya V. Pyatrikas, Alexei V. Stepanov, Anna V. Fedyaeva, Nina N. Varakina, Tatyana M. Rusaleva, Gennadii B. Borovskii & Eugene G. Rikhvanov

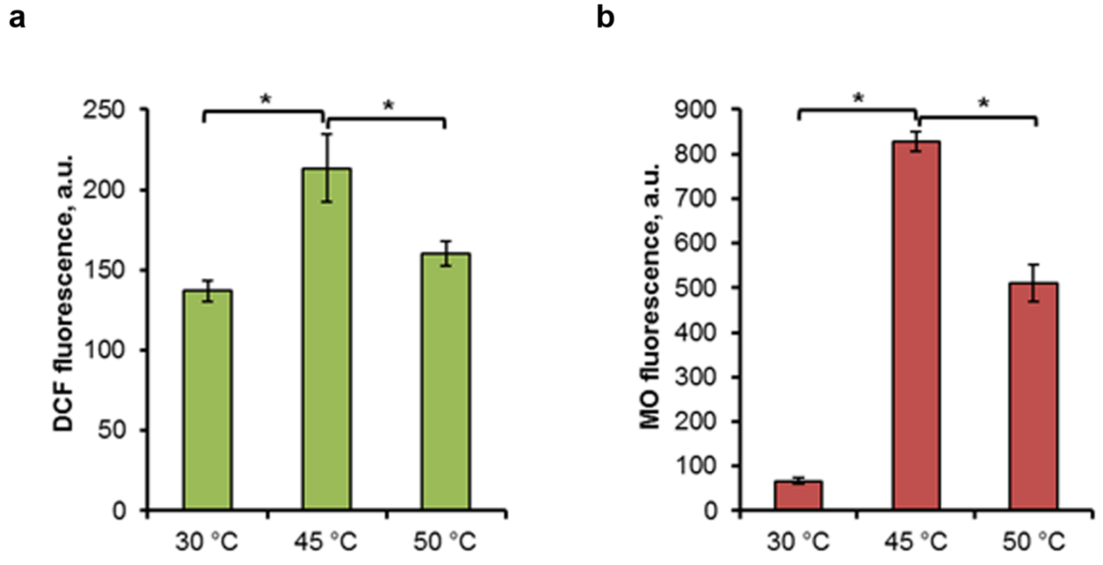

Figure S1. ROS production and MMP rise under moderate (45 °C) and hard (50 °C) heat shock. Cells of strain W303-1A were grown in YEPD medium and incubated at 30, 45 or 50 °C for 10 min. DCF (**a**) and MO (**b**) fluorescence were measured immediately after treatment. The data are the means of three or five independent experiments ± SE. *$p < 0.05$ (Student's two-tailed t-test).

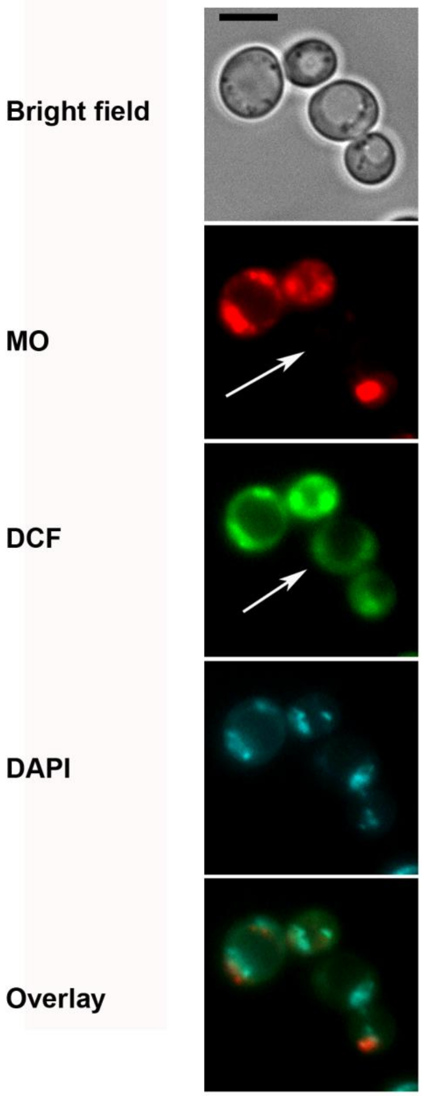

Figure S2. Intracellular localization of ROS in yeast cells under (45 °C) heat shock. Cells of parent type W303-1A were grown in YEPD medium for 24 h, double-stained with MO, DCF and DAPI (4,6-diamidino-2-phenylindole) and treated at 45 °C for 10 min. Microphotographs of yeast cells are presented. The scale bar is 5 µm. Arrows indicate cells with different MO and DCF staining. The Pearson's correlation coefficient between MO and DCF fluorescence for given image is 0.583.

.

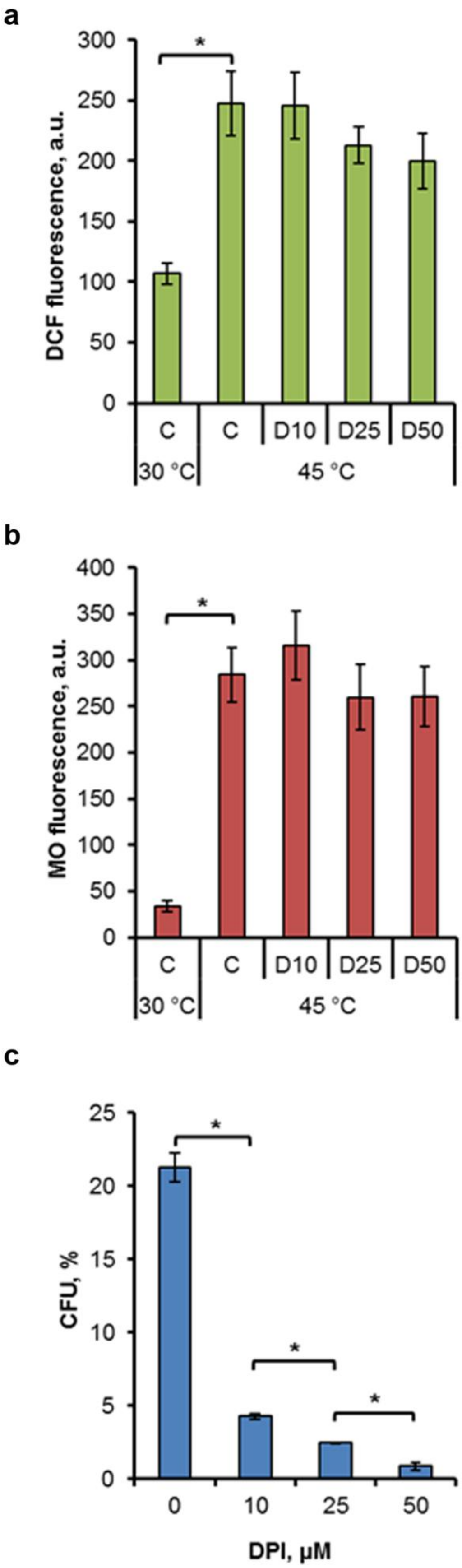

Figure S3. The effect of DPI on ROS generation, MMP rise and cell death under heat shock in the logarithmic growth phase of *S. cerevisiae* cells. Cells of parent type W303-1A were grown in YEPD medium and incubated at 45 °C in the presence of 0, 10, 25 or 50 μM DPI. DCF (**a**) and MO (**b**) fluorescence were measured immediately after 10 min treatment at 45 °C. Survival was evaluated by CFU counting (**c**) after 30 min treatment at 45 °C followed by 48 h incubation at 30 °C. The data are the means of three or four independent experiments ± SE. *< 0.05 (Student's two-tailed t-test).

a

b

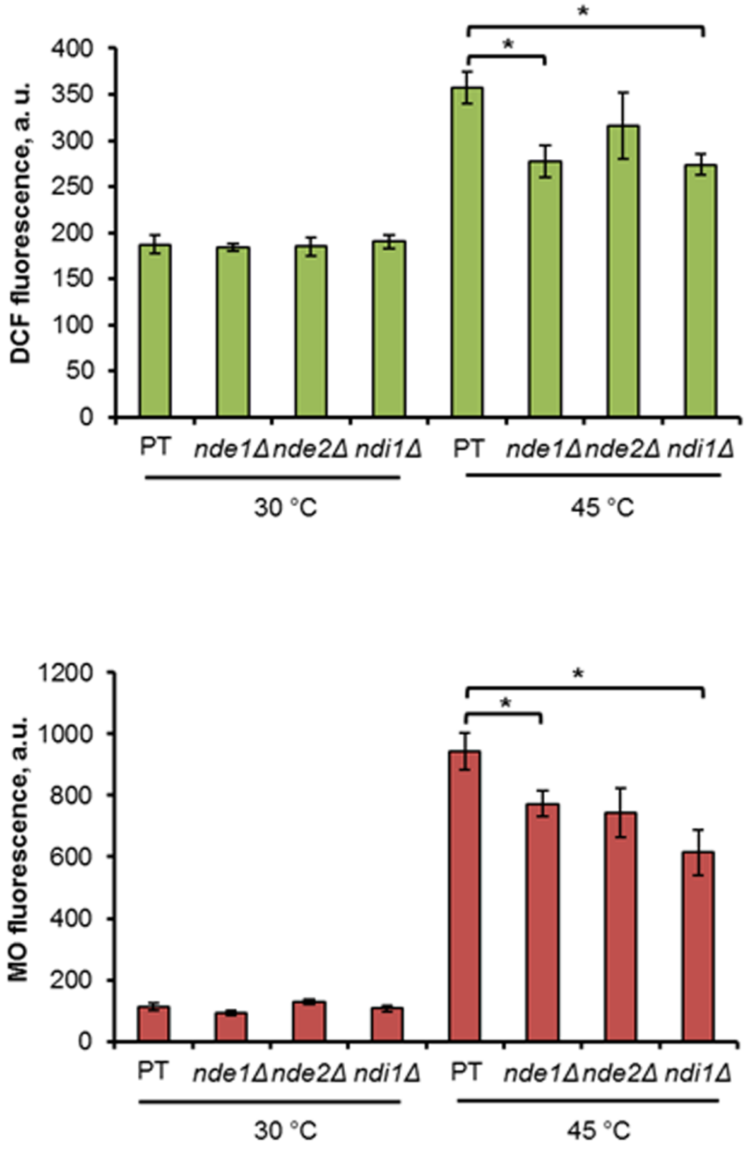

Figure S4. Heat-induced ROS generation and MMP rise in the logarithmic growth phase *S. cerevisiae* cells growing on galactose medium. Cells of parent type W303-1A (PT), single *nde1Δ*, *nde2Δ* and *ndi1Δ* mutants were grown in YEPGal medium for 4-5 h and incubated at 45 °C. DCF (a) and MO (b) fluorescence were measured immediately after 10 min treatment at 45 °C. The data are the means of three or four independent experiments ± SE. *$p < 0.05$ (Student's two-tailed t-test).

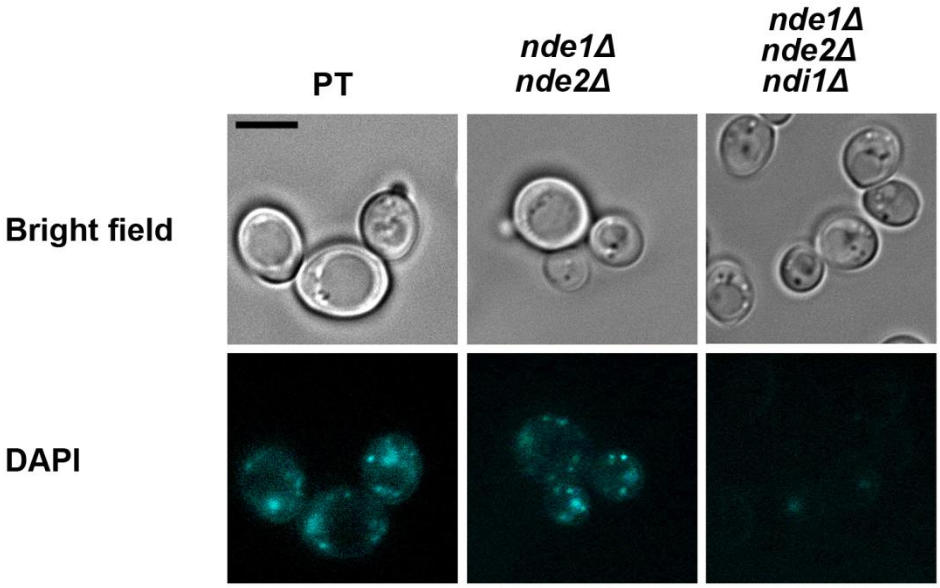

Figure S5. The DAPI staining of mitochondrial DNA in the cells of parent type, double *nde1Δnde2Δ* and triple *nde1Δnde2Δndi1Δ* mutant. Cells of parent type W303-1A (PT), double *nde1Δnde2Δ* and triple *nde1Δnde2Δndi1Δ* mutants were grown in YEPD medium and stained by DAPI at 30 °C for 10 min. Microphotographs of yeast cells are presented. The scale bar is 5 μm. 100 % of triple *nde1Δnde2Δndi1Δ* cells lost mitochondrial DNA.

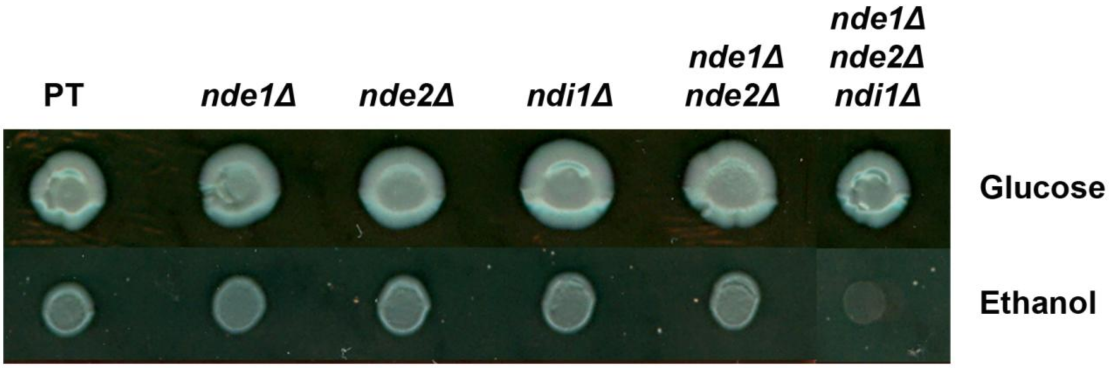

Figure S6. **Growth phenotype of mutants using non-fermentable carbon source.** Cells of parent type W303-1A (PT), single *nde1Δ*, *nde2Δ*, *ndi1Δ,* double *nde1Δnde2Δ* and triple *nde1Δnde2Δndi1Δ* mutants were spotted on YEPD or YEPE medium and grown for 48 h at 30 °C. The data is representative of three independent experiments.

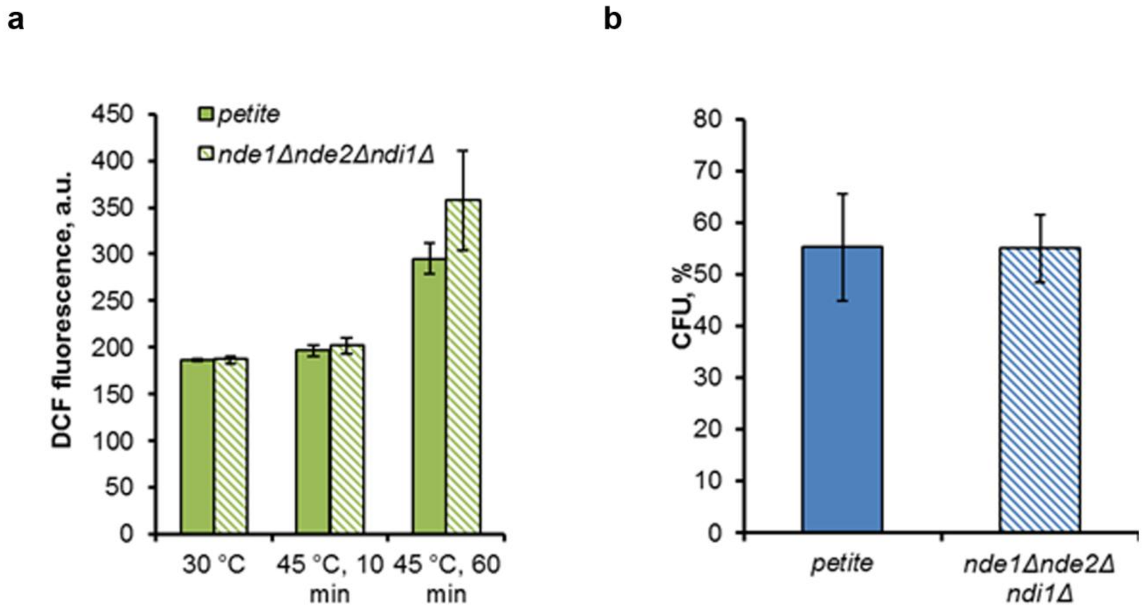

Figure S7. **Heat-induced ROS generation and heat-induced cell death in *petite* and triple *nde1Δnde2Δndi1Δ* mutant cells.** Cells were grown in YEPD medium for 24 h and incubated at 45 °C. DCF fluorescence (a) and survival (CFU counting) (b) were measured immediately after 60 min treatment at 45 °C. DCF was added to incubation medium for 10 or 60 min. The data are the means of four independent experiments ± SE.

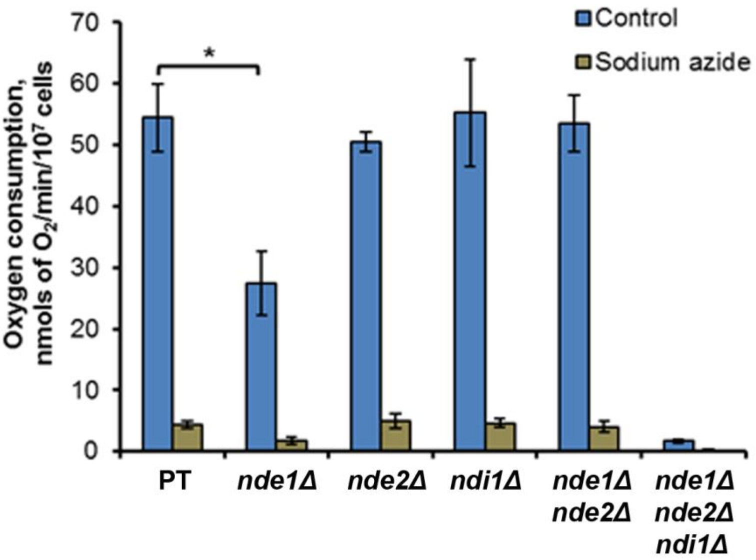

Figure S8. Respiratory activity in the cells of parent type, double *nde1Δnde2Δ* and triple *nde1Δnde2Δndi1Δ* mutants. Cells of parent type W303-1A (PT), single *nde1Δ*, *nde2Δ* and *ndi1Δ,* double *nde1Δnde2Δ* and triple *nde1Δnde2Δndi1Δ* mutants were grown in YEPD medium. Oxygen consumption rates were measured at 30 °C in the growth medium and expressed in nanomoles of O_2 per minute per 10^7 *S. cerevisiae* cells. Sodium azide (0.15 mM) was added to suppress respiration. The data are the means of four independent experiments ±SE. *$p < 0.05$ (Student's two-tailed t-test).

Assay of cellular respiratory chain activity. Cells were grown in YEPD medium up to the stationary phase (24 h). Yeast suspension (1.4 ml) was introduced into the thermostatic chamber, and oxygen consumption was assayed polarographically with a Clark-type electrode at 30 °C using the Hansatech Oxytherm system (United Kingdom). Oxygen consumption rates are expressed as nmol O_2 per min per 10^7 cells.

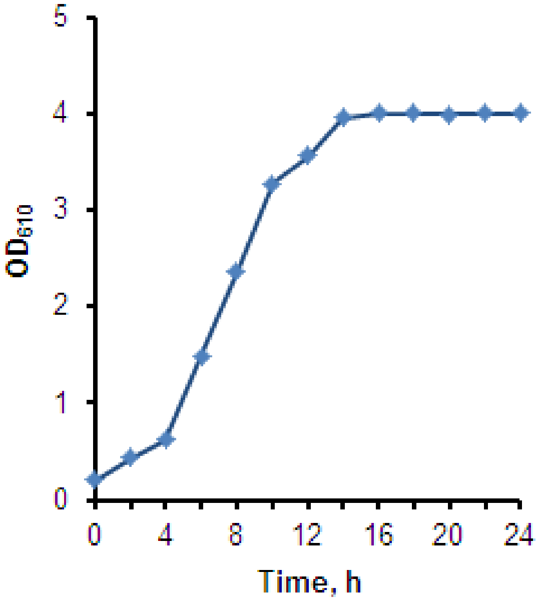

Figure S9. **Growth of parent W303-1A strain in YEPD medium.** Cultures (OD 0.1) of was inoculated in the liquid YEPD medium and grown at 30 °C for 24 h. Growth rates were determined by measuring optical density at 600 nm. The data is representative of three independent experiments.
